# Local and Systemic Cytokine Expression in Patients with Postherpetic Neuralgia

**DOI:** 10.1371/journal.pone.0105269

**Published:** 2014-08-15

**Authors:** Nurcan Üçeyler, Michael Valet, Waldemar Kafke, Thomas R. Tölle, Claudia Sommer

**Affiliations:** 1 Department of Neurology, University of Würzburg, Würzburg, Germany; 2 Department of Neurology, Klinikum rechts der Isar, Technische Universität München, München, Germany; University of Sao Paulo, Brazil

## Abstract

**Background:**

Postherpetic neuralgia (PHN) is the painful complication of a varicella zoster virus reactivation. We investigated the systemic and local gene expression of pro- and anti-inflammatory cytokine expression in patients with PHN.

**Methods:**

Thirteen patients with PHN at the torso (Th4-S1) were recruited. Skin punch biopsies were obtained from the painful and the contralateral painless body area for intraepidermal nerve fiber density (IENFD) and cytokine profiling. Additionally, blood was withdrawn for systemic cytokine expression and compared to blood values of healthy controls. We analyzed the gene expression of selected pro- and anti-inflammatory cytokines (tumor necrosis factor-alpha [TNF] and interleukins [IL]-1β, IL-2, and IL-8).

**Results:**

IENFD was lower in affected skin compared to unaffected skin (p<0.05), while local gene expression of pro- and anti-inflammatory cytokines did not differ except for two patients who had 7fold higher IL-6 and 10fold higher IL-10 gene expression in the affected skin compared to the contralateral unaffected skin sample. Also, the systemic expression of cytokines in patients with PHN and in healthy controls was similar.

**Conclusion:**

While the systemic and local expression of the investigated pro- and anti-inflammatory cytokines was not different from controls, this may have been influenced by study limitations like the low number of patients and different disease durations. Furthermore, other cytokines or pain mediators need to be considered.

## Introduction

Postherpetic neuralgia (PHN) is the painful complication of a varizella zoster virus reactivation. Up to 30% of patients with acute herpes zoster develop PHN resulting in persistent pain that is often resistant to analgesic treatment [1], [2]. The pathophysiology of pain in PHN is incompletely understood. High pain intensity during acute herpes zoster infection is one risk factor [3] and the question is why. One potential mechanism for the development of this localized pain syndrome with frequently associated severe allodynia and sometimes also itch [4] is the influence of algesic mediators such as pro-inflammatory cytokines, which might sensitize nociceptors locally.

Cytokines are pleiotropic small proteins that are key players in the induction and maintenance of neuropathic pain [5]. In several neuropathic pain conditions such as painful neuropathies, complex regional pain syndrome or small fiber neuropathy, an imbalance of systemic or local cytokine profiles has been reported e.g. for pro- and anti-inflammatory interleukins [6]–[9]. Although it is unclear if alterations in cytokine profiles are the cause or the consequence of neuropathic pain in these disorders, data from animal experiments give unequivocal evidence for the importance of cytokine homoeostasis as the basis for physiological pain perception [10]. In PHN, so far, only few and conflicting data on cytokine profiles are available. One study showed increased interleukin (IL)-8 concentrations in the cerebrospinal fluid of PHN patients [11], while in another study CSF levels of IL-8, IL-1α, IL-1β, IL-10, and tumor necrosis factor-alpha (TNF) were not different between patients with PHN and healthy controls [12]. In turn, long-term anti-TNF treatment (e.g. in patients with rheumatoid arthritis) while increasing the overall risk of herpes zoster, seems to reduce the incidence of PHN [13], [14].

Here we investigated if pain in PHN is associated with alterations in systemic and local cytokine profiles. We hypothesized that patients with PHN have a local and systemic pro-inflammatory cytokine profile compared to healthy controls and compared to unaffected skin.

## Methods

### Patients and healthy controls

Between 2007 and 2010, 13 patients with PHN localized in the dermatomes Th4 to S1 of the torso were recruited at the Departments of Neurology of the Universities of Würzburg and Munich, Germany. The group consisted of five men and eight women with a median age of 68 years (range 36-88). All patients were seen and examined by a neurologist and the diagnosis was confirmed. Routine examination included standardized assessment of pain and in addition of depressive symptoms that were analyzed with the “Allgemeine Depressionsskala” (ADS, German version of the Centre for Epidemiologic Studies Depression Scale) [15]. For blood cytokine analysis a control group of eleven healthy volunteers without infectious disease, history of PHN, or pain at the time point of blood withdrawal was recruited. The control group consisted of three men and eight women with a median age of 61 years (range 37–73). Due to the diversity of the lesion site in PHN patients we did not perform respective skin biopsies in healthy controls. Subjects were excluded in case of current infectious diseases, heavy physical activity or alcohol consumption the day before. Our study was approved by the Würzburg and Munich Medical School Ethics Committees. Study participants were included after written informed consent was obtained.

### Quantitative sensory testing

All patients underwent quantitative sensory testing (QST) according to the standardized protocol of the DFNS (“Deutscher Forschungsverbund Neuropathischer Schmerz”, German Research Network of Neuropathic Pain) [16]. Thermal and mechanical detection and pain thresholds were determined in the affected painful area in comparison to a contralateral unaffected area. In patients with the unaffected area in the back we also compared individual QST results with published normative values [17]. Parameters included cold and warm detection thresholds (CDT, WDT), thermal sensory limen (TSL), cold and heat pain thresholds (CPT, HPT), mechanical detection threshold (MDT), mechanical pain threshold (MPT), mechanical pain sensitivity (MPS), vibration detection threshold (VDT) and pressure pain threshold (PPT). Based on the log transformed raw values for each item a z-score sensory profile was calculated. The presence of dynamic mechanical allodynia was determined by gentile brush of the respective area.

### Blood withdrawal and RNA extraction

Five ml of venous whole blood was collected between 8 and 9 a.m. in PAXgene Blood RNA Tubes (PreAnalytics, BD, Hombrechtikon, Switzerland) after over-night fasting from all study participants. Blood samples were kept at room temperature for two hours before storage at −80°C. For RNA extraction the PAXgene Blood RNA MDx Kit (BD, Hombrechtikon, Switzerland) was used.

### Skin punch biopsy

A 5-mm skin punch biopsy (Stiefel GmbH, Offenbach, Germany) was taken in local anesthesia from the affected painful and a corresponding contralateral unaffected site of patients with PHN as previously described [9]. Each skin specimen was divided into two pieces: one piece was incubated in 4% paraformaldehyde (PFA) for two hours at +4°C and was further processed for protein gene product 9.5 (PGP 9.5) staining; one piece was put in RNAlater RNA Stabilization Reagent (Qiagen, Hilden, Germany) and stored at -80°C before RNA extraction.

### RNA extraction from skin samples

After thawing, the skin samples were homogenized in 1 ml TRIzol reagent (Invitrogen, Karlsruhe, Germany) and homogenized with an Ultraturrax homogenizer (Polytron PT 1600E, Kinematica, Luzern, Switzerland). After adding 200 µl of chloroform the samples were incubated at 25°C for 3 min and after spin down (12.000 g×15 min×4°C) the supernatant was mixed with 500 µl isopropanole. After incubation (25°C for 10 min) and another spin down (12.000 g×10 min×4°C) the pellet was washed with ethanol 75%. After another centrifugation step (7500 g×5 min×4°C) the samples were air-dried on ice for 5 min and the pellet was dissolved in water treated with diethylpyrocarbonate. Then the samples were incubated in a water bath at 55°C for 10 min.

### Reverse transcription PCR

For reverse transcription of 750 ng of the extracted RNA, TaqMan Reverse Transcription Reagents (Applied Biosystems, Darmstadt, Germany) were used. The reaction was carried out at a total volume of 100 µl. Five µl of random hexameres were added to 750 ng RNA and the mixture was filled up with RNase free water to an end volume of 37.8 µl. After heat denaturation (85°C, 3 min), 2 µl of Oligo-dt, 10 µl 10× PCR-buffer, 6.25 µl Multiscribe reverse transcriptase, 2 µl RNase inhibitor, 22 µl MgCl^2^, and 20 µl dNTPs were added. The PCR cycler conditions were as follows: annealing at 25°C for 10 min, reverse transcription at 48°C for 60 min and enzyme inactivation at 95°C for 5 min.

### Quantitative real-time PCR

Five µl of cDNA were used for quantitative real-time PCR (qRT-PCR), which was performed in the GeneAmp 7700 sequence detection system (Applied Biosystems, Darmstadt, Germany) capable of fluorescence using TaqMan Universal Master Mix (Applied Biosystems, Darmstadt, Germany). Gene specific oligonucleotide primers and probes for human in IL-1β, IL-6, IL-8, tumor necrosis factor-alpha (TNF), and IL-10 as well as the endogenous control 18sRNA were obtained as TaqMan Assays (Applied Biosystems, Darmstadt, Germany; see Table 1 for Assay-ID). The reaction contained 12.5 µl TaqMan Master Mix and 1.25 µl of the specific primer in an end volume of 25 µl. The cycler conditions were as follows: incubation for 2 min at 50°C followed by another incubation step at 95°C for 10 min, afterwards 45 cycles with 15 sec at 95°C and 1 min at 60°C. In order to guarantee primer specificity and to exclude genomic contamination, negative controls without cDNA template were run on each qRT-PCR well plate. All samples were measured as triplicates, while the 18s-values were measured as duplicates.

**Table 1:** Characteristics of the patient group.

#	Age [yrs]	Gender	Time since pain onset [yrs]	Affected body area	IEND affected area [fibers/mm]	IENFD unaffected contralateral area [fibers/mm]	Allodynia	Current pain intensity [NRS]	ADS score
1	69	F	15	Right thoracic Th5	2	28	no	8	13
2	79	M	4	Left paravertebral Th6	19	17	no	3	5
3	64	F	20	Right lumbosacral L5	0	6	no	5	37
4	36	M	3	Left paravertebral Th4	5	10	no	7	refused
5	62	F	4	Left paravertebral Th4	5	8	no	4	9
6	75	M	7	Left axillary Th4	1	6	yes	3	19
7	59	F	5	Left subaxillary Th4	5	5	yes	8	31
8	53	F	1	Left parasacral	7	15	yes	5	39
9	88	M	0.5	Left ventral Th5	0	8	no	8	refused
10	72	F	1	Left ventral Th4	2	19	yes	8	7
11	68	F	0,2	Left dorsal Th12	18	29	yes	7	12
12	70	F	0.1	Left thoracic Th12	21	26	yes	4	17
13	47	M	0.1	Right back L5	16	24	no	8	24

**Abbreviations:**

ADS: “Allgemeine Depressionsskala” (German version of the Centre for Epidemiologic Studies Depression Scale); F: female; M: male; IENFD: intraepidermal nerve fiber density; NRS: numeric rating scale from zero to ten (zero: no pain; ten: worst pain imaginable); yrs: years.

For gene expression analysis in blood samples each qRT-PCR reaction plate contained a calibrator sample, which was the blood sample of the control person whose threshold cyclus (Ct)-values (i.e. qRT-PCR cyclus at which a significant signal was detected) were next to the calculated mean of all control blood samples specific for each primer. The evaluation of the obtained data was performed with the comparative ΔΔCt-method and data of PHN patients were compared with healthy controls. For gene expression analysis in skin samples the gene expression in the affected skin sample of the individual patients were compared with the gene expression in the respective unaffected skin sample. Again the ΔΔCt-method was used.

### PGP 9.5 immunofluorescence for intraepidermal nerve fiber density

After fixation in 4% PFA (pH 7.4) for two hours at 4°C, skin samples were washed in 0.1 M phosphate buffer and transferred to 10% sucrose over-night. Afterwards tissue was embedded in Tissue Tek, frozen in 2-methylbutane cooled in liquid nitrogen and was stored at −80°C before further processing. Fifty-µm sections were cut with a microtome and were stained with rabbit polyclonal antibodies to human PGP 9.5 (Ultraclone, UK, 1∶800; primary antibody) and goat anti-rabbit IgG labeled with cyanine 3.18 fluorescent probe (Amersham, USA, 1∶100; Cy3, secondary antibody). A microscope (Axiophot 2, Zeiss, Jena, Germany) with a CCD camera (Visitron Systems, Tuchheim, Germany) and SPOT advanced software (Windows Version 4.5) were used for visualization. Nerve fibers were counted using a 40× objective according to standard criteria [18]. The average intraepidermal nerve fiber density (IENFD) per mm of epidermal length was calculated following international standards [19].

### Assessment of inflammatory cells in the skin

We used 10 µm skin sections to visualize T-cells (CD-3, 1∶200, Abcam, Germany), macrophages (CD-68, 1∶3000, Abcam, Germany), and Langerhans cells (CD-1a, 1∶500, Beckman Coulter, Krefeld, Germany). Stains were performed using a standard immunohistochemistry protocol (ABC kit, Vector, Germany) with diaminobenzidine as chromogen and hemalaun as a counterstain. Cells were assessed semiquantitatively by an investigator blinded to the diagnosis with regard to cell density: 0 = no cells; 1 = single cells; 2 = several cells; 3 = dense cell infiltrates.

### Statistical analysis

Data were analyzed using IBM SPSS software (Version 21, Ehningen, Germany). qRT-PCR data did not display normal distribution in the Shapiro-Wilk-test, thus we used the Mann-Whitney-U-test for comparison of independent groups; the QST data were normally distributed and the t-test for independent groups was applied. We used the Levene test to check for equality in variance. Statistical significance was assumed at p<0.05.

## Results

### Characteristics of study population

Table 1 gives baseline data of the PHN patients. Seven patients were on analgesic medication with pregabalin (n = 5), amitriptyline, carbamazepine, duloxetine, fentanyl, flupirtine, lidocaine, and tilidine (n = 1 each) as monotherapy or in different combinations. The median duration of pain was four years (four months to 20 years) and the median current pain intensity on a numeric rating scale (0 = no pain, 10 = worst pain imaginable) was 7 (3–8). No correlation was found between depressive symptoms and pain.

### Patients with PHN have hypoesthesia for warm and touch

**Figure 1:**
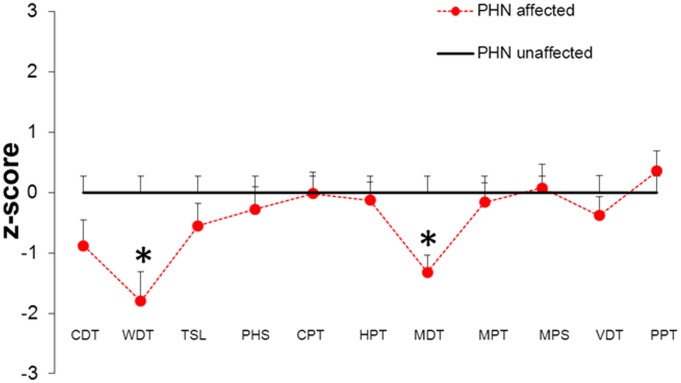
Sensory profile of patients with postherpetic neuralgia (PHN) assessed with quantitative sensory testing (QST), affected skin is compared to unaffected skin (zero line). In affected skin, patients showed elevated detection thresholds for warm and for tactile stimuli (*p<0.05). Abbreviations: CDT: cold detection threshold; CPT: cold pain threshold; HPT: warm pain threshold; MDT: mechanical detection threshold; MPS: mechanical pain sensitivity; MPT: mechanical pain threshold; PHS: paradoxical heat sensation; PPT: pressure pain threshold; TSL: thermal sensory limen; VDT: vibration detection threshold; WDT: warm detection threshold.

QST showed that warm detection (p<0.05) and detection of mechanical stimulation (p<0.05) were impaired in the affected skin area compared the unaffected side in patients with PHN (Fig. 1). Dynamic mechanical allodynia was present in 6/13 (46%) patients in the affected area. QST parameters did not correlate with pain. Also, QST results of the unaffected contralateral area at the back (n = 5 patients, see Table 1) were not different from published normative values.

### Cutaneous innervation is reduced in affected skin

**Figure 2:**
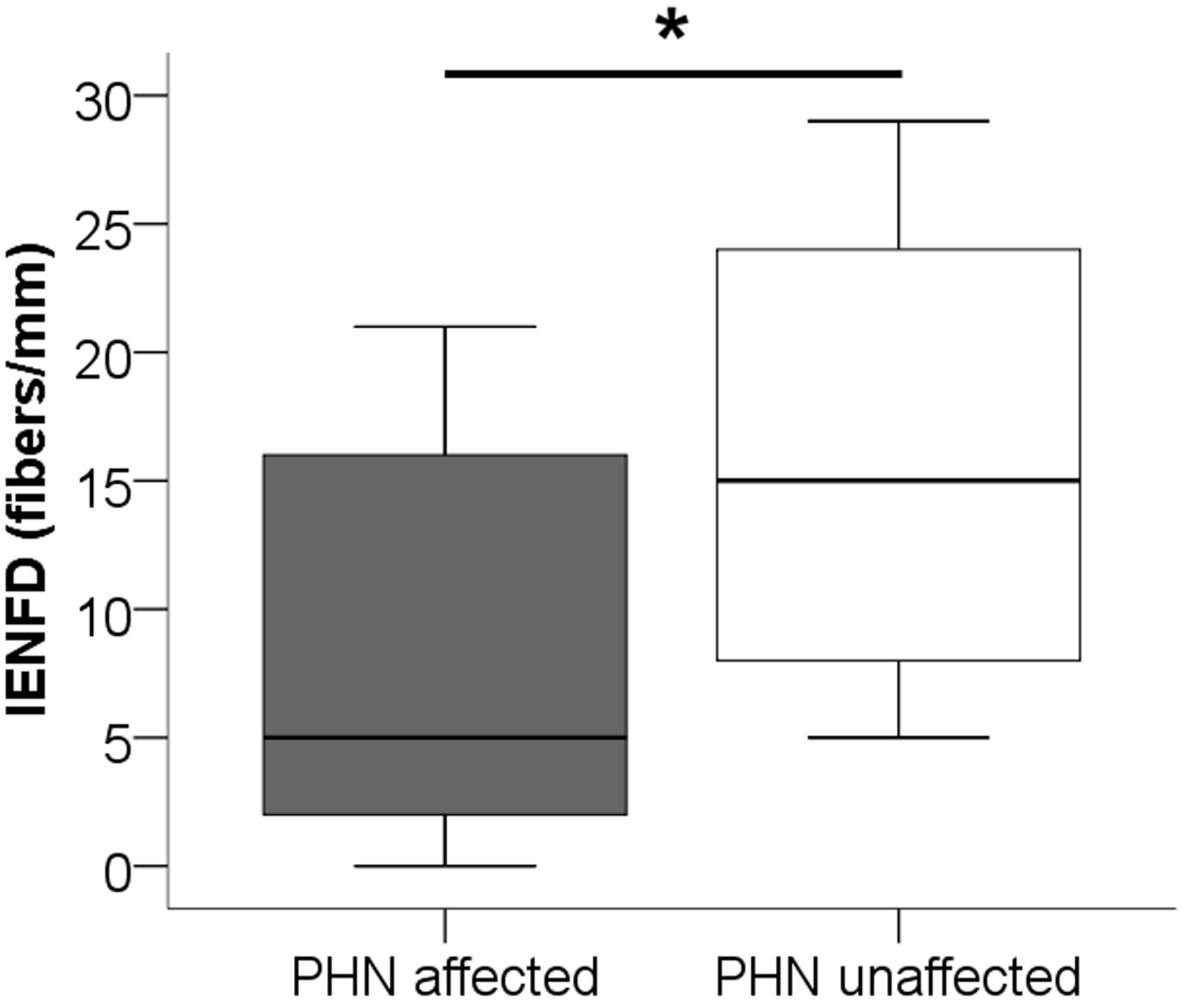
Boxplots show the intraepidermal nerve fiber density (IENFD) in affected versus unaffected skin of patients with postherpetic neuralgia (PHN). In affected skin IENFD is lower than in corresponding (contralateral) unaffected skin (*p<0.05). The horizontal black line in the box marks the median value.

In 11/13 (85%) patients with PHN the IENFD was lower in skin of the affected site compared to the unaffected contralateral side (p<0.05; Fig. 2). The median IENFD in affected skin was 5 fibers/mm (0–21 fibers/mm) and in unaffected skin 15 fibers/mm (5–29 fibers/mm). IENFD did not correlate with pain.

### No inflammatory infiltrates are present in affected an unaffected skin

The semiquantitative assessment of dermal T-cells and macrophages and epidermal Langerhans cells did not reveal cellular infiltrates in the affected and unaffected skin (data not shown).

### Systemic cytokine gene expression does not differ from controls

**Figure 3:**
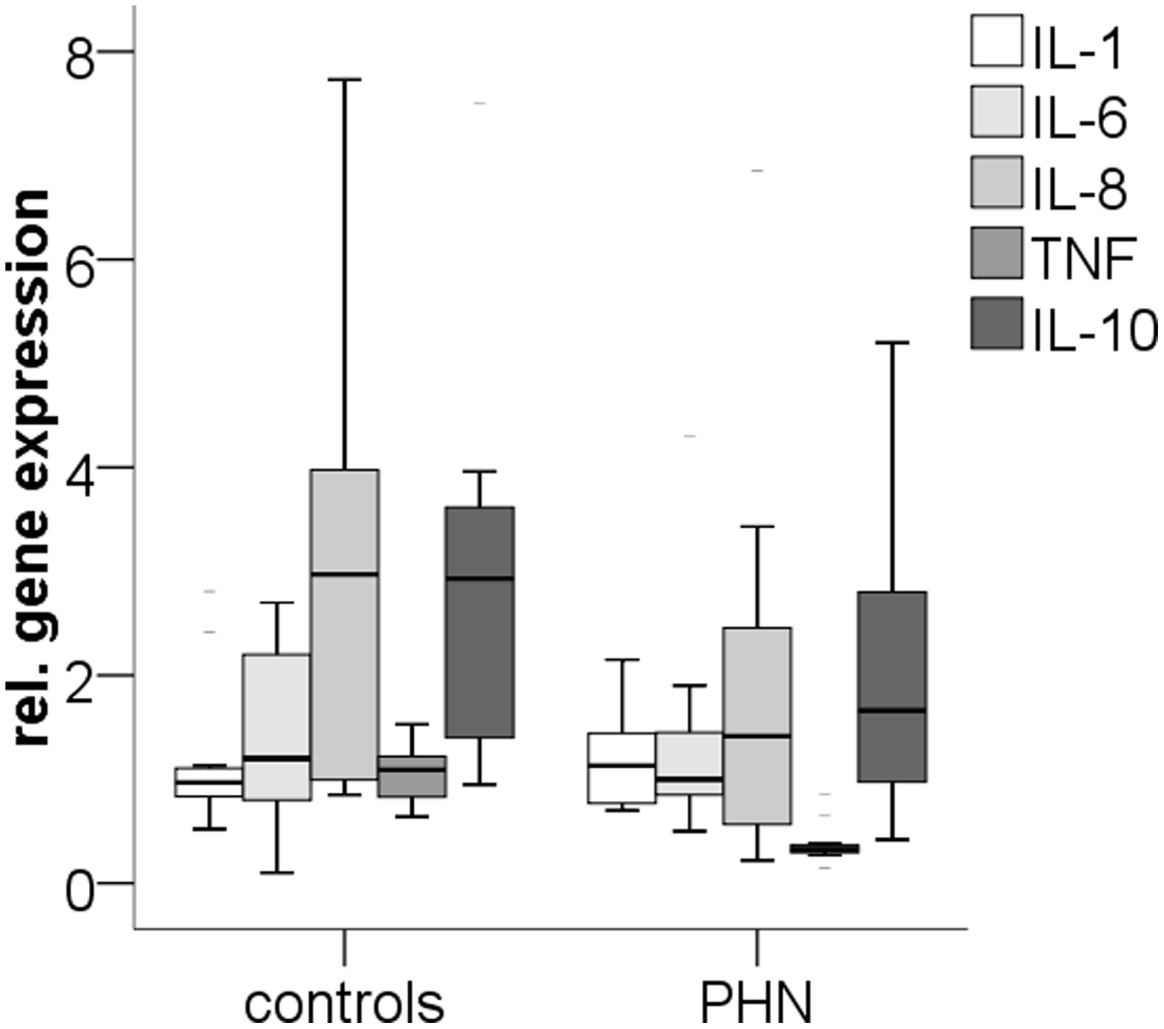
Boxplots illustrate the systemic (blood) relative gene expression of the pro- and anti-inflammatory cytokines interleukin-1β (IL-1β), IL-6, IL-8, tumor necrosis factor-alpha (TNF), and IL-10 comparing patients with postherpetic neuralgia (PHN) and healthy controls. No intergroup difference was found.

Systemic gene expression of the pro- and anti-inflammatory cytokines IL-1β, IL-6, IL-8, TNF, and IL-10 did not differ between patients with PHN and healthy controls (Fig. 3). Also, no difference was found when comparing patients with and without current analgesic medication.

### Local cytokine gene expression does not differ between affected and unaffected sides

**Figure 4:**
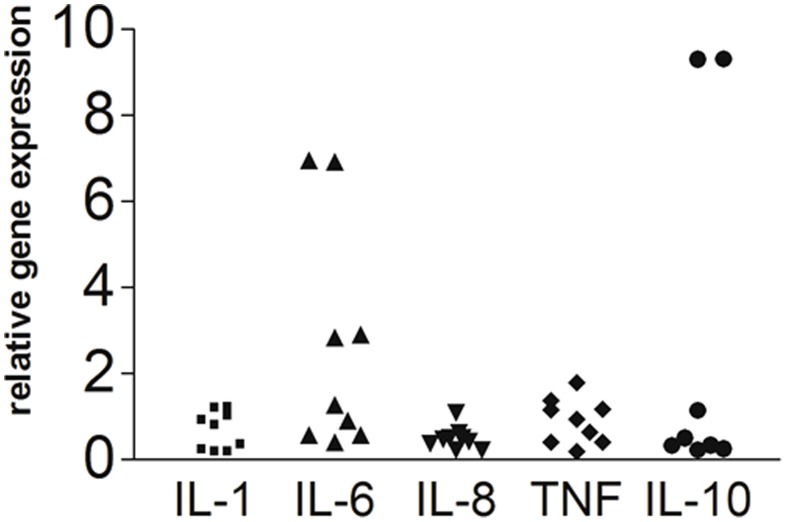
Scatter plots illustrate the local (skin) relative gene expression of the pro-inflammatory cytokines interleukin-1β (IL-1β), IL-6, IL-9, tumor necrosis factor-alpha (TNF) and the anti-inflammatory cytokine IL-10 in affected skin of patients with postherpetic neuralgia (PHN) compared to unaffected (contralateral) skin that is calibrated to a relative gene expression of 1.

Local gene expression of the investigated markers was similar in affected and unaffected skin of patients with PHN (Fig. 4) for all investigated cytokines irrespective of current analgesic treatment. Also, patients with and without allodynia did not differ in cytokine expression patterns. In two patients IL-6 and IL-10 gene expression was very high in the affected skin (7fold higher IL-6 expression and 10fold higher IL-10 expression compared to unaffected skin area; patients #5 and #9 in Table 2).

**Table 2:** Primers and Assay-IDs of the TaqMan assays used.

Primer	Assay-ID
IL-1β	Hs00174097_m1
IL-6	Hs00174131_m1
IL-8	Hs00174103_m1
TNF	Hs00174128_m1
IL-10	Hs00174086_m1

**Abbreviations:**

IL: interleukin; TNF: tumor necrosis factor-alpha.

## Discussion

PHN is associated with severe localized pain of incompletely understood pathophysiology. We confirm the reduction of IENFD in affected skin compared to unaffected skin [20], [21], however, in contrast to our hypothesis, we did not observe a pro-inflammatory cytokine profile in PHN patients. No changes were found in systemic and local cytokine gene expression profiles of patients with PHN compared to healthy controls and between affected and unaffected skin.

### Cytokine measurements in PHN

Cytokines play a crucial role in neuropathic pain induction and maintenance [5], [10]. This has been shown in a large number of different animal models and also in several human neuropathic pain disorders. Especially in localized pain states such as small fiber neuropathy and complex regional pain syndrome elevated cytokine levels have been reported [9], [22]. A local increase in algesic pro-inflammatory cytokines was therefore assumed in PHN, however, it had not been studied so far.

In patients both with acute herpes zoster and PHN, cytokine levels have been investigated in the cerebrospinal fluid (CSF) [11], [12] and in the blood [23] giving conflicting results. In the study by Kotani et al. control subjects had an IL-8 concentration of 18 µg/L (7 to 24 µg/L) in the CSF compared to 81 µg/L (57 to 380 µg/L) in patients with herpes zoster during rash and after rash healing (44 µg/L, 28 to 101 µg/L). An initially increased IL-8 CSF level was found to be an independent predictor of PHN [11]. In another study IL-1α, IL-1β, IL-10, TNF, MIP-1a, and fractalkine levels were below the detection limit in the majority of the investigated 25 PHN patients similar to controls and IL-8 levels were not different between groups [12]. This discrepancy might be explained by the fact that in the study by Rijsdijk et al. a PHN cohort had been examined that had a median pain duration of 24 months, thus IL-8 levels may have normalized in the meantime. Similarly, our patients had a median duration of pain of four years, and we cannot preclude that there might have been differences in cytokine expression earlier on. However, cytokine expression in our cohort did not correlate with disease duration (data not shown).

Zhu et al. showed that in serum samples of patients who already suffered from pain in the acute phase of herpes zoster IL-1β, IL-6, IL-8, TNF, and IL-10 protein levels were higher compared to healthy controls. In particular, IL-6 protein levels were higher in ten of the investigated 49 patients who later developed PHN and positively correlated with pain severity [23]. The observational period of this study was six months.

The assumption that changes in cytokine expression levels may be of pathophysiological importance in PHN induction and maintenance is supported by observations in patients that received anti-cytokine medication. In a retrospective analysis of patients with rheumatoid arthritis on anti-TNF treatment the number of PHN after herpes zoster was remarkably lower (only two cases out of 206) compared to data of the general population [13]. In contrast, the development of severe PHN was reported after inadvertent infliximab administration [24]. In a prospective study assessing the risk of herpes zoster in more than 5000 patients with rheumatoid arthritis treated with anti-TNF drugs, 86 episodes of herpes zoster were recorded in 82 patients; in two of these patients PHN occurred [14].

In another study Zak-Prelich assessed serum cytokine levels and different subtypes of skin lymphocytes in patients with herpes zoster who did and did not develop PHN [25]. Interestingly, a reduced number of cellular infiltrates was found at the lesion sites of herpes zoster patients that developed PHN compared to those who did no without association with systemic cytokine levels. The authors suggested that an impaired immune response upon zoster infection resulted in an impaired containment of infection and more damage of the dermatome as the basis for neuralgia. Here, we did not find infiltrates of T-cells and macrophages in affected skin of PHN patients compared to unaffected skin, which may be due to the different duration of disease in our study until biopsy, while in the study by Zak-Prelich et al. patients were seen at standardized time points (within the first ten days of disease and one week later).

### Treatment of PHN

Since part of the pathology in PHN has been assumed to be located in the skin itself, also surgical excision of the lesioned skin has been tried. However, this approach did not lead to sustained pain relief [26], which indicates that the assumed pathology might be located upstream the pain pathway such as in dorsal root ganglia neurons. Interestingly, a recent case report described the analgesic effect of local laser therapy of the skin over a period of 13 months in a 73 year old women who had been suffering from PHN for 15 years [27]. The author suggested that the reducing effect of laser on local cytokine expression might be the underlying mechanism of this observation, however, cytokine levels were not investigated. In another case report the analgesic effect of anti-IL-2 that lasted throughout the three years of the follow-up period was described in a patient with PHN [28], but again cytokine levels were not measured before and after treatment.

### Affected skin is denervated, unaffected skin is spared

Histological assessment of skin samples also revealed denervation of affected skin compared to unaffected contralateral skin in patients with PHN. This finding is in accordance with pervious findings. Petersen et al. reported a 40% reduction in painful skin compared to painless skin [21]. Similar results were found in the study of Buonocore et al. [20]. In accordance with these studies we also did not find a correlation between IENFD and the presence or absence of allodynia. As outlined in Table 1, IENFD of patients with and without allodynia ranged from normal to complete denervation. Also, no correlation was found between IENFD and patients' current pain intensity. Further studies are needed to decipher the underlying mechanisms that may range from hyperexcitable remaining nerve fibers causing allodyina while IENFD is normal, to hyperactive dorsal root ganglion neurons independent of peripheral nerve fiber density.

### Limitations

The major limitation of our study is the relatively low number of patients and controls. Although we were recruiting at two centers, only 13 patients with PHN were seen during the study period of three years. This may be due to the fact that in the majority of cases patients with PHN are not hospitalized but treated as outpatients. Our patient group was inhomogeneous with regard to disease duration, which may have influenced our results such that we missed early changes in cytokine gene expression. We cannot exclude influence of current analgesic medication on the study results since the study cohort was small and those patients on analgesic medication used drugs from different pharmaceutical groups. We focused on cytokines as major algesic mediators, and due to limitations in bio-samples only a limited number of targets could be investigated. Also, no protein measurements were possible. Besides cytokines the assessment of further algesic mediators (e.g. chemokines, opioid receptors, neurotrophic factors) would have been desirable.
